# Cystic nephroma: a case report and review of the literature

**DOI:** 10.1186/1757-1626-1-267

**Published:** 2008-10-23

**Authors:** Konstantinos Stamatiou, Konstantinos Polizois, Gerasimos Kollaitis, Stefanos Dahanis, Grigoris Zafeiropoulos, Christos Leventis, Theocharis Lambou

**Affiliations:** 1 Department of Urology, Thriasio Hospital, Elefsina, Greece

## Abstract

**Background:**

The spectrum of cystic renal neoplasms includes both benign and malignant tumors and the order is as follows: benign multilocular cyst, multilocular cystic renal cell cancer and cystic renal cell cancer. Gross similarities among multicystic tumors of the kidney may cause conflict in the diagnosis and treatment of these lesions.

**Results:**

We report a 53-year old male patient who presented with a mild persistent left flank pain and a painful left renal mass. After a series of examinations including abdominal ultrasound, intravenous pyelography and computed tomography, he underwent radical nephrectomy. Microscopic examination of the resected tissue showed the typical characteristics of a cystic nephroma. Immuno-histological staining of the epithelium of the tumour with CK 19 suggested an aberrant renal tubular differentiation.

**Conclusion:**

Cystic nephroma is a relatively rare benign lesion of the kidney. Since 1892, only 200 cases have been reported in the international literature. The non-specific clinical findings and the poor contribution of imaging examinations make the preoperative distinction from other cystic renal neoplasias difficult. Final diagnosis can be established in the histopathological examination of the completely rejected tumor in the pathology laboratory.

## Background

Cystic nephroma (CN), also called multilocular cystic nephroma, is a rare, non-genetic, benign, renal cystic lesion. It is usually present as a unilateral, multicystic renal mass without solid nodules. According to the "WHO classification of the renal neoplasm's" it is grouped along the mixed epithelial-stromal tumor of the kidney [1]. Histologic features include: cysts lined by flat, cuboidal, or hobnail epithelium and septa variably lined by fibrous and/or ovarian-like stroma. While the histologic features of cystic nephroma are well described, gross similarities with other cystic tumours of the kidney and especially with cystic renal cell carcinoma may cause confusion in the diagnosis and conflict in the treatment of this lesion [1].

## Case reports

**Figure 1:**
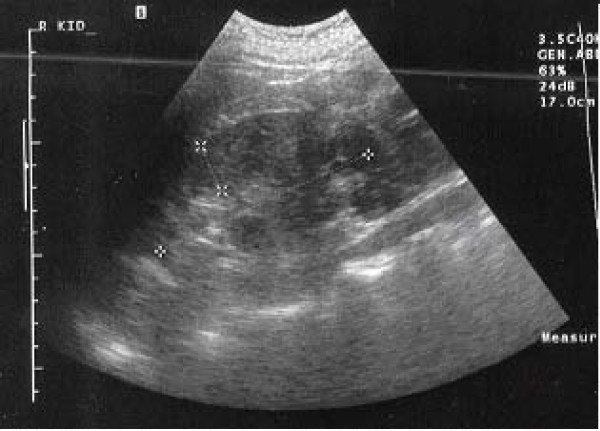
Cystic nephroma: Renal ultrasound image.

**Figure 2:**
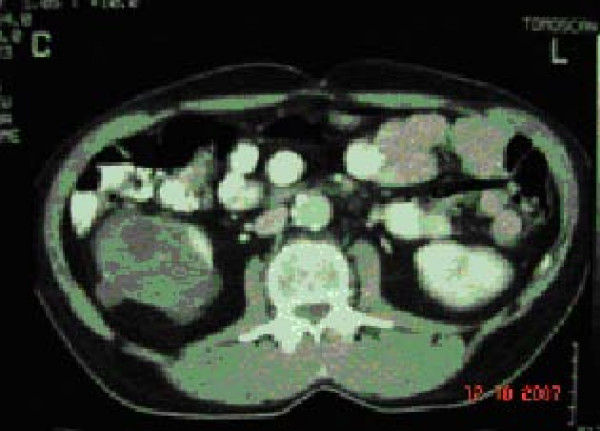
Cystic nephroma: Computed tomography image.

**Figure 3:**
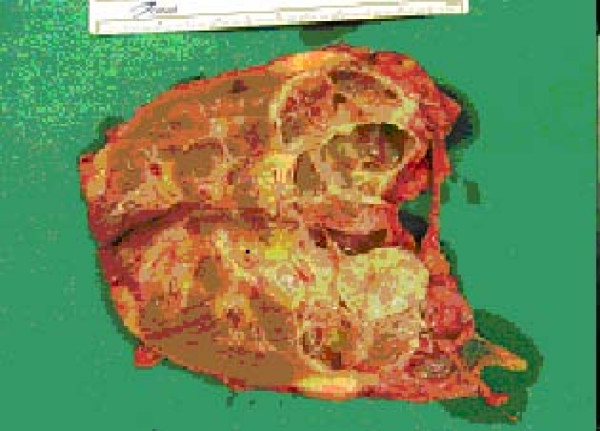
Cystic nephroma: Macroscopic appearance.

**Figure 4:**
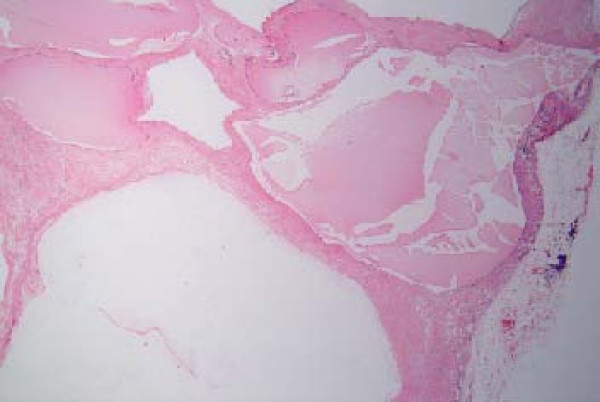
Cystic nephroma: Microscopic appearance.

**Figure 5:**
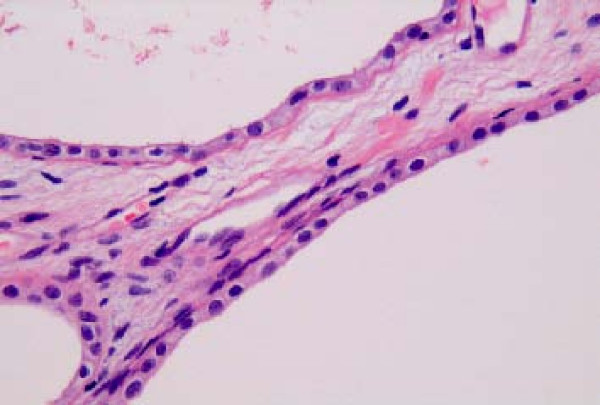
Low cuboidal or epithelium of the cyst wall.

**Figure 6:**
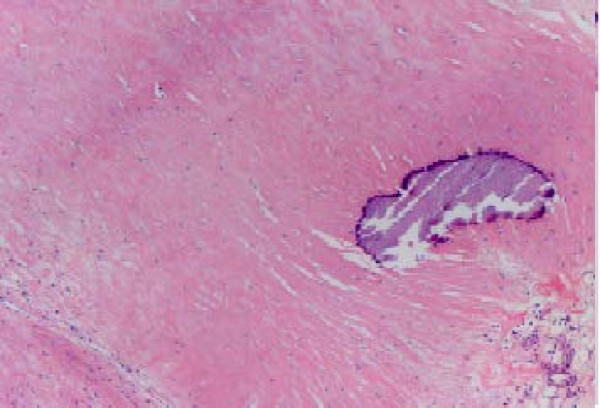
Area with metaplastic ossification.

**Figure 7:**
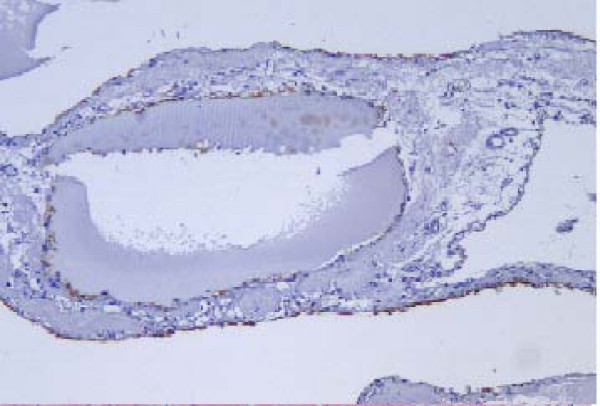
Immuno-histological staining of the epithelium of the tumour with CK 19.

**Figure 8:**
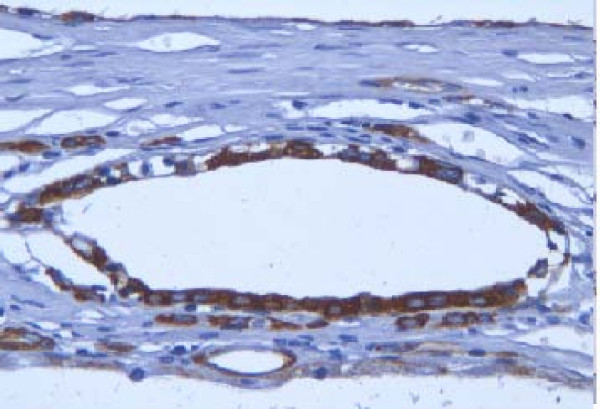
Immuno-histological staining of the epithelium of the tumour with CK 19.

A 53-year old male patient was presented to the outpatient department with a mild persistent right flank pain. No history of right colicky pain or macroscopic hematouria was reported. Physical examination did not reveal tenderness or palpable mass at the right costo-vertebral angle. Laboratory findings were normal. Ultrasound scanning revealed a multicystic mass having multiple thick septae within the mass, closely related to the pelvicalyceal system (Fig. 1). Doppler ultrasound showed no vascularity within the mass. Excretory urogram demonstrated dilatation of the right renal pelvis and lower calyces. Computed tomography of the abdomen displayed a multilocular cystic mass of 7 cm in diameter, showing a sharp interface with adjacent renal parenchyma with no wall thickening, calcification, or enhancement, at the lower pole of the right kidney (Fig. 2). No lymphadenopathy and metastatic disease were noted. Despite the relatively benign radiologic appearance of the mass, a radical nephrectomy was carried out. The renal specimen was weighed 371 gram and measured 7 × 6 × 6 cm. Grossly, renal external surface was unremarkable while the cut surface revealed numerous cysts at the lower pole pushing the renal pelvis (Fig. 3). The cysts were filled with serous fluid and a rim of normal renal tissue was recognized within the tumour. Microscopic examination showed the typical characteristics of a cystic nephroma with multiple cysts lined by low cuboidal or flattened epithelium (Fig.4 and 5), with bland nuclear chromatin and were separated by fibroblastic stroma. The wall of the cysts also revealed areas with metaplastic ossification (Fig. 6). Accumulations of inflammatory cells were observed in the wall of the renal pelvis. Immuno-histological staining of the epithelium of the tumour with CK 19 suggested an aberrant renal tubular differentiation (Fig. 7 and 8).

## Discussion and conclusion

Edmunds reported the first case of CN in 1892 as cystic adenoma of the kidney [2]. Less than 200 cases have been reported up to date in the international literature but this number should be regarded with caution because of the variety of terms used to describe this entity (cystic renal hamartoma, cystadenoma, polycystic nephroma etc) [3,4]. Currently this is the third case of CN published in the Greek literature up to date [5,6].

Multilocular cystic nephromas could be both congenital, affecting predominantly infant males, as well as acquired affecting predominantly postmenopausal females [7]. The congenital form is usually seen in infants under the age of two, with a male to female ratio of 2:1 and is generally unilateral, although bilateral lesions are reported [8,9]. In contrast, in adults it is usually seen between 4^th ^and 6^th ^decade, and has a male to female ratio of 1:9 [4]. While it is clear that adult CN shares common morphologic characteristics with congenital CN, several authors consider it as a separate entity showing completely different biologic behaviour [10]. This belief is based upon reports of congenital CN cases with nodules of Wilms tumor among the benign cysts [4]; however, malignant changes in adult CN cases can occur as well [11]. The exact aetiology of both congenital and adult CN is unresolved, while the classification is controversial. According to Eble and Bonsib, CN stands at one end of a spectrum of renal cystic diseases of the childhood that includes pure CN, cystic – partially differentiated nephroblastoma, multilocular cysts with nodules of Wilms tumor and Wilms tumours [8]. Similarly, in adults (as in our case) the order would be CN, multilocular cystic renal cell carcinoma and cystic renal cell carcinoma. Generally, it is believed that CN is malformation that enlarges by fluid accumulation and cystic dilatation of individual locules and present as tumor-like cystic mass [12]. Some investigators suggested this entity to be classified among the mixed epithelial stromal tumors, while others consider it as a separate entity [13]. According to the "WHO classification of tumours" [1], CN is classified as a special entity that belongs or is identical to mixed epithelial and stromal tumours (MESTK) of kidney, especially when ovarian type stroma or stroma with elements like white or fibrous bodies is present [1]. The pathogenesis of these tumours seems to be based on the influence of hormones since they mostly affect women, especially those with history of oral intake of estrogens. Moreover, in rare reported cases of male patients with mixed epithelial and stromal tumor, there was a history of hormone manipulations for the treatment of prostate cancer [14]. The presence of estrogen and progesterone receptors in the stroma of these tumours further supports the abovementioned hypothesis [15]. However, a history of hormone therapy is not present in all male patients (as in our case) while, some of reported cases lacked evidence of hormonal receptor expression [16,17]. Reviewing the above, it is reasonable to conclude that not all cases implicate hormonal mechanism in pathogenesis and therefore the expression of hormone receptors in the ovarian-like stroma of cystic nephroma is probably related to ontogenic similarity to ovarian stroma and smooth muscle differentiation.

Diagnostic criteria established by Eble and Bonsib include: an expansible mass surrounded by a fibrous pseudo capsule whose interior is composed entirely of cysts and septa, with no expansible solid nodules. The cysts should be lined by flattened, cuboidal, or hobnail epithelium. The septa may contain epithelial structures resembling mature renal tubules but should not contain epithelial cells with clear cytoplasm and should be free of skeletal muscle fibbers [8].

CN presents with no-specific urinary tract symptoms such as abdominal or flank pain, urinary tract infection symptoms, hematuria and hypertension in adults and as palpable abdominal mass in childhood [18]. CN presents with no-specific imaging findings as well. Plain radiographs may show a mass, rarely with calcification. The excretory urogram usually demonstrates a well-defined, intra-renal mass in a normal functioning kidney. Delayed excretion with hydro-calycosis or no visualization occurs in cases with obstruction by pelvic herniation of the tumor. Sonographic findings relate to the size of the locules. When locules are small, a non-specific complex intra-renal mass is demonstrated. In contrast, when locules are large the sonogram will demonstrate a renal mass with multilocular configuration, discrete septa and sono-lucent spaces. Computed tomography usually reveals a smooth multilocular mass and determines its perinephric extend. However, the discrimination between category II and III cysts according to the Bosniak system for the radiological diagnosis of cystic renal lesions is often difficult [18,19]. Color-doppler flow imaging is suggested as a useful tool for the differential diagnosis of malignant versus benign lesions: on angiographic examination, CN is usually hypo vascular and less commonly shows no vascularity at all. MRI angiography is an alternative in preoperative evaluation for possible partial nephrectomy.

The non-specific clinical findings and the poor contribution of imaging examinations render the exact preoperative distinction from other cystic renal neoplasias difficult. In fact, final diagnosis can be established in histopathological examination of the completely rejected tumor in the pathology laboratory. Boggs and Kimmelstiel defined certain criteria in order to enable the differentiation from polycystic disease, multicystic kidneys, simple renal cysts and cystic renal cell carcinoma. These criteria include: multilocular lesion, cysts lined with epithelium, cysts that do not communicate with the pelvis and normal residual renal tissue. While the histologic features of CN are well described [1], final pathologic diagnosis is almost exclusively based on immunohistochemistry. In the presence of atypical epithelial cells lining the cysts and when the nuclear features of the clear cells in the septa are misleadingly bland, the positivity for vimentin and EMA not aid in the distinction of CN from cystic renal cell carcinoma. In such cases, stromal positivity for estrogen or progesterone receptors may be helpful however, since a substantial proportion of cases are ER/PR-negative, absence of staining does not preclude the diagnosis of cancer [14].

Notably, several investigators reported that – as in our case – CN cyst epithelium shows positivity for CK, suggesting an aberrant renal tubular differentiation [4]. This co-expression of factors of proximal and distal renal tubules in the epithelium of cysts has also been reported in several other renal tumours.

## Conclusion

Our case highlights the already known differentiation of the cyst epithelium towards the epithelium of proximal and distal renal tubules and the absence of any differentiation towards stromal elements. All of the above suggest the opinion of separating CN from MESTK. As for the treatment of these lesions, since the preoperative diagnosis between CN and cystic renal cell carcinoma is impossible, nephrectomy seems to be the most preferable treatment. Despite the benign nature of CNs, limited experience in the diagnosis and treatment pose the need for systematic follow up imperative.

## List of abbreviations

MESTK: Mixed epithelial and stromal tumours; CN: Cystic nephroma.

## Consent

The authors state that written informed patient consent was obtained for publication of the report and the accompanying images.
